# A Retrospective Analysis of Factors Affecting Palliative Care Consults in Patients Undergoing Cytoreductive Surgery and Hyperthermic Intraperitoneal Chemotherapy

**DOI:** 10.7759/cureus.12589

**Published:** 2021-01-09

**Authors:** Robin R Rodriguez, Laila Babar, Herman Lo, Obaid Ashraf, Dulabh Monga, Gene Finley, Lisa Doverspike, Amber Blackledge, Ashish Sethi, Moses S Raj

**Affiliations:** 1 Medical Oncology, Allegheny Health Network, Pittsburgh, USA; 2 Medical Oncology, The University of Iowa Hospitals and Clinics, Iowa City, USA; 3 Internal Medicine, Allegheny Health Network, Pittsburgh, USA; 4 Hematology and Oncology, Allegheny Health Network, Pittsburgh, USA; 5 Palliative Care, Butler Health System, Butler, USA

**Keywords:** palliative and supportive care, gi oncology, hyperthermic intraperitoneal chemotherapy, barriers to consultation, quality of life (qol), cytoreductive surgery

## Abstract

Purpose

This study was conducted to determine factors that influence palliative care (PC) consultation in patients receiving cytoreductive surgery (CRS) with hyperthermic intraperitoneal chemotherapy (HIPEC).

Patient and methods

We queried our Electronic Medical Record EPIC for a list of patients who underwent cytoreductive surgery with HIPEC or hyperthermic intrathoracic chemotherapy (HITEC) in the hospital from April 2016-April 2019. Data was manually extracted and patients who did not meet our criteria were excluded. Patients were divided on the basis of palliative care consults and differences between the groups were analyzed. Odds ratios (OR) with p-value of 0.05 and confidence interval of (CI) 95% were calculated.

Results

We identified 55 patients of whom 34 met our inclusion criteria: 11 males and 23 females with an average age of 56 years at the time of diagnosis. Eight patients (23%) had PC, with six having commercial insurance, seven married, and six with more than one comorbid medical issue. Comorbidities >1 (OR: 0.12; CI: 0.02-0.76; p: 0.02) and age >40 (OR: 0.015; CI: 0.0007-0.3029; P: 0.006) were associated with a higher likelihood of PC. Gender, insurance type, and marital status did not have a significant association with PC. Mean age between PC consulted patients versus non-PC consulted patients was 58.5 vs. 55.9 and median age between the two groups was 60.5 vs. 60 which also showed a trend towards higher rates of PC in the older population.

Conclusion

Approximately one quarter of patients who underwent CRS with HIPEC had a concurrent PC consult. Though this is better than the national average of 11-16%, it continues to be a very small number. Efforts must be made to engage PC early in the course of treatment and recognize it as an integral part of cancer care. PC is not only an end-of-life service, in fact, studies have shown that early consultations lead to higher patient satisfaction, improved quality of life, and better communication.

## Introduction

The National Cancer Institute defines palliative care (PC) as care given to improve the quality of life (QOL) for patients with life-threatening diseases [1]. As life-threatening illnesses inclusive of cancer affect not only the body of the patient but also the mindset and family dynamics, palliative care seeks to offer both a physical and emotional response that is unique to the needs of each patient. In 2014, the World Health Assembly Resolution on Palliative Care called for all countries to incorporate PC but there have still been limitations on its incorporation even in some of the healthcare systems in the United States [2].

According to research by Hawley, “nearly one-third of US hospitals with more than 50 beds do not have any palliative care service”. Some reasons cited for why palliative care has not become universal include lack of resources, fear for patient’s mindset, ignorance as to what is palliative care, and considering palliative care consultation as admittance of failure [3]. However, the role of PC is ever evolving. As more studies are being conducted, its role in the improvement of QOL for patients is being widely recognized. In all life-threatening illnesses, it has been consistently demonstrated that PC plays an integral role in overall comfort for not only the patients but their families as well [4]. Thus, the significance of PC should be understood, especially in advanced stage cancer patients.

Cytoreductive surgery, followed by treatment with hyperthermic intraperitoneal chemotherapy (HIPEC) is an option for patients that have either local or metastatic cancers of the abdomen including gastric, colon, ovarian, and pseudomyxoma peritonei, among others [5]. Cytoreductive surgery (CRS) with HIPEC is not standard of care for patients with abdominal malignancies; however, it is considered on a case by case basis for patients with advanced stage IV abdominal cancers with peritoneal carcinomatosis [6]. The two-step process begins with surgical resection of all visible peritoneal tumors followed by the insertion of a peritoneal catheter in which a heated chemotherapy agent is injected directly into the abdominal cavity [5]. Although initially considered to cause a higher risk of morbidity and mortality, research supports that the morbidity and mortality rate of CRS with HIPEC are similar to those of other abdominal oncologic surgeries. However, this procedure is still rather controversial [7]. The associated morbidity complications are often divided into both surgery related and chemotherapeutic morbidities. These risks range from fistulas, anastomotic leaks, leukopenia, and organ toxicity among others [8]. Morbidity and mortality rates of CRS with HIPEC, described by Chua et al., range between 12-52% for morbidity and 0.9-5.8% for mortality [9]. The inherent morbidity and mortality of the disease itself calls for the expertise of palliative medicine specialists independent of CRS with HIPEC. Nevertheless, given the increased morbidity of patients receiving CRS with HIPEC as well as the fact that this procedure is only offered to advanced stage cancer patients, it is logical to believe that they would receive the greatest benefit from palliative care services.

## Materials and methods

For this retrospective analysis, the electronic medical record system EPIC of the Allegheny Health Network (AHN) was queried for patients that had been consulted, evaluated, treated or who were considered for treatment with CRS and HIPEC between April 2016 and April 2019. Because the purpose of this study was to elucidate potential variables affecting a clinician’s decision to consult palliative care, numerous parameters were obtained. These parameters included the following: gender, age, diagnosis, cancer type, metastasis, time from diagnosis to HIPEC, 30- and 90-day outcome, any hospitalization 30 days, 90 days and one year after surgery, family history, chemotherapy treatment, insurance plans, past medical history of cancer, one year outcome, and comorbidities. These factors were then compared to determine any correlation for patients who received PC consult and those who did not.

Inclusion criteria incorporated patients who were treated or were considered for treatment with CRS and HIPEC. Patients that had the conditions which met the surgical procedure standard but who were not considered for treatment with CRS and HIPEC were excluded from this study. Thirty-four patients met the criteria for the research project.

## Results

Of these 34 patients, 23 were female and 11 were male. The average age at diagnosis was 56 years. Only eight received palliative care consults. Of these eight, seven were female and one was male, average age was 59 years. The median age between those who received PC versus those who did not was 60.5 years versus 60 years, respectively (Table 1).

**Table 1:** Characteristics of palliative care consulted (PC) patients CRS: Cytoreductive surgery; HIPEC: Hyperthermic intraperitoneal chemotherapy.

No.	Gender	Age	CRS HIPEC	Marital Status	Insurance	1-Year Outcome		Comorbidities
1	Female	50	Yes	Married	Aetna	Deceased		>3
2	Female	61	Yes	Married	Tricare	Alive		>3
3	Female	62	Yes	Married	BCBS	Deceased		2
4	Female	43	Aborted	Married	BC	Deceased		>3
5	Female	64	Aborted	Divorced	Med Assist	Unknown		>3
6	Female	60	Aborted	Married	PPO	Deceased		>3
7	Male	59	Aborted	Married	Community	Deceased		2
8	Female	69	Yes	Married	Medicare	Deceased		>3

The mortality rate was higher in those that received PC consults. For the 90-day outcome, six out of eight patients were still alive, with one being deceased and the other patient’s status unknown. For the one-year outcome, six out of the eight patients were deceased; one was alive while the other patient’s status was still unknown. Of the patients who received PC, 75% were deceased within a year. In those patients that did not receive palliative care consults, the one-year outcome was 20 patients who were alive, four deceased, and the other two had unknown statuses. Thus, out of the patients who did not receive palliative care consults, only 15% were deceased one year after hospitalization.

Comorbidities were also found to be high in the group that received PC. Out of the eight patients that had palliative care consults, six of them were known to have three or more comorbid conditions, whilst the other two patients had two comorbid conditions. The average number of comorbidities in all 34 patients was 2.5.

Demographics were also considered. Only one of the eight patients had Medicare while the other seven patients had differing types of commercial insurance. Out of the eight PC patients, seven were married while the remaining one patient was divorced.

In comparing the aforementioned factors within the two groups (those who had PC versus those who did not), some positive associations were determined (Table 2). Patients with age greater than 40 years and those with more than one comorbidities were more likely to receive PC consultation (age: OR: 0.015; CI: 0.0007-0.3029; P: 0.006, comorbidities: OR: 0.12; CI: 0.02-0.76; p: 0.02). Sex, insurance, and marital status had no significant correlation in determining variables that affected PC consultation.

**Table 2:** Statistical analysis of palliative care (PC) consulted patients

Characteristic	Odd Ratio	95% Confidence Interval	p-value
Age >40 vs <40	0.015	0.007-0.3029	0.006
Comorbidities >1 vs <1	0.12	0.02-0.76	0.02
Sex: Male vs Female	0.229	0.00243-2.15	0.1964
Insurance: Government vs Commercial	0.750	0.123-4.56	0.755
Marital Status: Married vs Single/Divorced	0.388	0.04-3.74	0.412

Another factor which was identified and interesting to note was that half of the patients who received palliative care consults were unable to have HIPEC performed and only partial debulking of their tumor due to extensive metastasis. Thus, out of the eight patients, half of them had unsuccessful CRS with HIPEC procedures as their cancer was too far metastasized in the peritoneal cavity. Interestingly, out of the other 26 patients in this study only two other patients were unable to have their surgery completed due to extensive metastasis and both of these patients were deceased prior to one year following the intended surgery date.

## Discussion

Globally, only 14% of the population that needs palliative care is actually receiving it. In the United States, this patient population accounts for approximately 6 million who could benefit from palliative care [2]. Research shows palliative care and early PC consults improve the overall wellbeing of patients.

In a 2010 study reported in the New England Journal of Medicine, a cohort of patients with metastatic non-small cell lung carcinoma showed increased benefit in quality of life and decreased risk of depression when introduced early to the concept of palliative care [10]. Other studies have corroborated the significance of not only introducing palliative care during hospitalization but also introducing PC early, not only for the sake of the patient but for the family members as well [4].

However, there is a certain stigma around palliative care that has to do with a lack of knowledge as to what palliative care actually entails. Palliative care is often confused with hospice care [11]. This misinformation contributes to the hesitancy of obtaining a palliative care consult as some providers believe that it means they have failed their patients [3]. On the same note, this misunderstanding of palliative care also makes some patients distrustful and uninterested in learning more as they feel that acceptance of PC is acceptance of end of life. Some patients have expressed the thought that accepting a PC consult is a way for the healthcare system to lessen costs and provide substandard care [11]. This is clearly a fault that needs to be corrected. It needs to be understood that although hospice is a branch of palliative care, they are not mutually exclusive.

In an era marked by an ever-growing field of cancer research, particularly with the advent of immunotherapy, palliative care has become even more important. Patients with cancers such as melanoma, which were previously seen as ‘incurable’, are now living longer lives and with every new breakthrough in research, we are seeing overall survival improve [12]. In this environment it is imperative that we value quality of life just as highly as quantity of life. Additionally, we are learning about ways to better prognosticate upon our patients’ disease courses, and can also use these prognostic markers to guide our approach to palliative care consultation and management [13].

Palliative care should be a priority for all patients who are battling life-threatening illnesses and not only for those who are considered to be at the end of life. In a paper by Meritens et al., 145 oncology providers were surveyed and out of the respondents, 51% felt that palliative care consultations were underutilized in their hospital. However, 42% of these respondents also felt that palliative care should only be consulted when the patient had less than six months to live [14]. In our cohort, the eight patients that were seen by PC, 75% were deceased within a year, in comparison to the 15% of those who did not have a PC consult. In correlation, the overall likelihood of having PC consultation increased with older age and more than one comorbidities. This is important to note as this lends to the idea that consultation was given to those with higher associative risk factors. As mentioned previously, CRS with HIPEC surgery is only considered in patients with advanced stage IV cancers which in and of itself should be enough for consideration of PC consultation. Thus, the erroneous ideology that PC is only end of life care is potentially present amongst AHN consulting providers as well. PC consults were seemingly placed for patients considered to be more seriously ill and/or to be fast approaching end of life. This error in thought is seemingly the largest confounding barrier to only eight out of the 34 patients in our study garnering a PC consult.

Despite numerous studies with proven benefits of early PC consultation, hospital systems are grossly underutilizing the service. CRS with HIPEC is a known aggressive treatment option for advanced stage cancers with controversy surrounding its use. Any possible associated increase in morbidity and mortality are nominal issues considering that any patient with a life-threatening illness benefits from receiving PC services. These patients would certainly benefit from their expertise in symptom management. Unfortunately, only a quarter of patients in our study received the benefits of PC. Although, above the national average of 11-16% for PC consults we recognize the small sample size may have positively affected the data.

Misconceptions of PC and conflation of PC and hospice will continue to be a barrier to patients receiving PC, unless more aggressive educational intervention is taken by hospital systems to consulting providers, and providers to patients and their families. A quick Google search of palliative care demonstrates the misinformation that forms the public’s opinion. Often palliative care is discussed as hospice care and for many patients that rely on internet sites for medical advice, it is noteworthy that something needs to be done at the hospital level in order to combat this misperception.

Palliative care not only improves QOL of patients, it reduces hospital costs as well. Aligning with this is a study conducted by Mount Sinai/Trinity College in 2018, showing that on average palliative care was associated with cost savings of $4,251 dollars per patient with cancer, and $2,105 for patients without cancer who received PC. It also showed early PC consult improved QOL [15]. Clearly, palliative care is not only beneficial for the patients and their families but also for the hospital system.

Realizing that the aforementioned misconception of palliative care and hospice care being synonymous as one of the most important barriers, it is important to try to assess what can be done in order to improve the patient’s ability to gain a PC consult when experiencing a life-threatening illness. Thus, some actions that have been considered to combat barriers to obtaining palliative care include using a hospital wide checklist criterion that would determine whether a patient should receive PC. According to Perrin and Kazanowski, this approach can increase consultation rates from 41% to 82% and decrease 30-day rehospitalizations from 36% to 17% [11]. Aside from decreased rate of hospitalizations, the use of a criterion that is hospital wide would definitely prove to be beneficial especially when it comes to the fact that a barrier of receiving PC is dependent upon an individual provider’s belief in PC. This would ensure that these biases would not affect a patient’s ability to gain a PC consult and would take off some of the burden of making the provider decide on whether a PC consult is warranted.

In addition, it is important to note that hospitals that do increase the use of palliative care consultations should also realize that with increased utilization of this specialty there will be an increase in demand for palliative care specialists. To fill this void, it needs to be considered how to attract more physicians and medical professionals into palliative care services. Along with this, there should also be increased attention in providing education for providers in how to handle discussing palliative care with patients. A greater emphasis on education of the healthcare system on how to approach this topic and the important impacts it could have on QOL could positively alter how it is conveyed to the patient and thereby lead to increased amounts of consultations for those patients who it would benefit.

## Conclusions

In summary, the purpose of this retrospective study was to determine factors which could affect palliative care consultation in patients receiving aggressive surgical treatment (HIPEC) for advanced stage cancers. Although it was determined that multiple variables such as gender, marital status, and insurance did not play a role in whether or not a patient received a PC consult, it was evidenced that PC consults were given to those patients who were older in age, had a greater number of comorbidities and subsequently had worse outcomes. This supports other research that illustrates one of the major barriers in obtaining a PC consult comes from the misperception of what a PC consult actually entails and what palliative care actually is. This study demonstrates that there needs to be reform at the hospital level to not only educate patients but providers in palliative care. Implementation of a hospital-wide criterion for determining eligibility is one way to effectively combat potential variables in who should receive PC consults.
